# Still water run deep: Therapeutic TP effect of ucMSC‐Ex via regulating mTOR to enhance autophagy

**DOI:** 10.1111/jcmm.18120

**Published:** 2024-02-15

**Authors:** Zhirong Zhao, Li Han, Mei Xin, Lichen Zhou, Kexin Jiang, Qian Huang, Ruiwu Dai

**Affiliations:** ^1^ Research Institute of General Surgery Jinling Hospital, Affiliated Hospital of Medical School, Nanjing University Nanjing Jiangsu Province China; ^2^ General Surgery Center General Hospital of Western Theater Command Chengdu Sichuan Province China; ^3^ College of Medicine Southwest Jiaotong University Chengdu Sichuan Province China; ^4^ Clinical Medical College Chengdu Medical College Chengdu Sichuan Province China; ^5^ Pancreatic injury and repair Key laboratory of Sichuan Province General Hospital of Western Theater Command Chengdu Sichuan Province China

**Keywords:** autophagy, exosomes, high‐throughput sequencing, mTOR, pancreatitis, stem cells

## Abstract

Our previous study confirmed that umbilical cord mesenchymal stem cells‐exosomes (ucMSC‐Ex) inhibit apoptosis of pancreatic acinar cells to exert protective effects. However, the relationship between apoptosis and autophagy in traumatic pancreatitis (TP) has rarely been reported. We dissected the transcriptomics after pancreatic trauma and ucMSC‐Ex therapy by high‐throughput sequencing. Additionally, we used rapamycin and MHY1485 to regulate mTOR. HE, inflammatory factors and pancreatic enzymatic assays were used to comprehensively determine the local versus systemic injury level, fluorescence staining and electron microscopy were used to detect the effect of autophagy, and observe the expression levels of autophagy‐related markers at the gene and protein levels. High‐throughput sequencing identified that autophagy played a crucial role in the pathophysiological process of TP and ucMSC‐Ex therapy. The results of electron microscopy, immunofluorescence staining, polymerase chain reaction and western blot suggested that therapeutic effect of ucMSC‐Ex was mediated by activation of autophagy in pancreatic acinar cells through inhibition of mTOR. ucMSC‐Ex can attenuate pancreas injury by inhibiting mTOR to regulate acinar cell autophagy after TP. Future studies will build on the comprehensive sequencing of RNA carried by ucMSC‐Ex to predict and verify specific non‐coding RNA.

## 1 INTRODUCTION

Traumatic pancreatitis (TP) is a special type of acute pancreatitis (AP) which occurs after abdominal trauma.^1^ Although TP has a low frequency in abdominal trauma, it causes excessive release of zymogen by pancreatic acinar cells, induces irreversible pancreatic injury and is highly susceptible to patient death.^2^, ^3^ In order to reduce the pancreatic tissue damage after TP and improve the follow‐up pancreatic acinar cells repair, our previous study confirmed that umbilical cord mesenchymal stem cells‐exosomes (ucMSC‐Ex) exert reparative effects on TP damaged pancreatic tissues by attenuating the inflammatory response through inhibiting the expression of inflammatory factors.^4^ Furthermore, most importantly we demonstrated that ucMSC‐Ex inhibit apoptosis of pancreatic acinar cells to exert protective effects.^5^ However, current research focuses on exosome mediated functional repair and tissue remodelling, and we convince that the research of exosomes should be multilevel, interdisciplinary and comprehensive.^6^ Further research is required to determine the specific mechanisms by which exosomes exert therapeutic effects and the interactive effects on the regulation of cellular life activities.

Macroautophagy (this will be referred to simply as ‘autophagy’ in this study) is well recognized as a mutual antagonism for apoptosis in the cellular vital movement.^7^ In autophagy, the misfolded proteins and lipids could be orderly degraded and further reused by the cell. Mammalian pancreatic tissue has a significantly higher autophagic flux than other tissues, and typical autophagosomes have been observed early in pancreatic acinar cells from experimental animal AP models and AP patients.^8^, ^9^, ^10^ Further intervention experiments found that disrupting genes encoding autophagy initiation processes blocks autophagy in the pancreas and induces spontaneous pancreatitis.^11^ Impaired autophagy in AP experimental animals directly leads to enhanced apoptosis and necrosis of pancreatic acinar cells and aggravates AP.^12^, ^13^ At present, scholars focus on regulating autophagy for treatment in AP.^14^ In autophagy, mTOR is the most important upstream of regulation, and researchers can significantly regulate autophagic flux by precise regulation of mTOR phosphorylation. One study achieved the goal of treating AP by inhibiting mTOR using rapamycin in a rat AP model, and the autophagy was observed to be significantly enhanced in pancreatic acinar cells.^15^ However, the relationship between apoptosis and autophagy in TP has rarely been reported, and further exploration of the autophagic effect of pancreatic acinar cells will be helpful to deeply investigate the specific mechanism of hucMSC‐Exs in the treatment of TP.^16^

An increasing number of studies are uncovering the genetic basis of multiple phenotypic traits through comprehensive analysis of whole transcriptome resequencing data.^17^ Analysis of transcriptome data can help to reveal the effects of experimental variables on cellular life activities at the gene expression level.^18^ In this study, we performed whole transcriptome resequencing of pancreatic tissue from TP rats after ucMSC‐Ex treatment to identify significantly altered cellular activities and target gene expression. Subsequently, we confirmed that ucMSC‐Ex exerted therapeutic effects after TP by enhancing autophagy through the regulation of mTOR.

## 2 MATERIALS AND METHODS

### 2.1 Whole transcriptome sequencing of rat pancreatic tissue

Total RNA was extracted from tissue samples. The concentration and purity of the extracted RNA were checked using nanodrop2000. RNA integrity was checked by agarose gel electrophoresis, and the values of Rin were determined using Agilent 2100. Eukaryotic mRNA has the structure of a Ploy‐A tail. The base pairing of A and T used magnetic beads with oligo (dT). mRNA was isolated from total RNA for analysis of transcriptome information. Adding to the fragmentation buffer, mRNA can be randomly fragmented and small fragments around 300 bp isolated by magnetic bead screening. Under the action of reverse transcriptase, six base random primers (random hexamers) were added, and mRNA was used as the template to reverse synthesize the first strand cDNA, followed by the second strand synthesis, which formed a stable double strand structure. The double stranded cDNA was end sticky, which was complemented with blunt ends by adding end repair mix at the 3 end for ligation of a Y‐shaped linker. Illumina 6000 platform is used for sequencing. All the gene data were analysed by PCA between samples. In addition, the KEGG databases were used to explore the differentially expressed genes and make statistical analysis.

### 2.2 Experimental materials

Sprague Dawley (SD) rats, weighing 200–250 g, were purchased from Chengdu Dashuo Experimental Animal Co., Ltd. (animal licence No.: SCXK [Chuan] 2020‐030). The multifunctional animal impact equipment has been authorized patent (self‐developed, patent number: ZL 2016 10347341.5).^19^ ucMSC‐Ex were provided by the Chengdu KangErmei Biological Cell Preparation Center (Number: G01210001). The enzyme‐linked immunosorbent assay (ELISA) kits for serum amylase, lipase and rat IL‐6, IL‐10, TGF‐β and TNF‐α were purchased from Shanghai Jiancai Biological Technology Co., Ltd. LC3B, mTOR, P62, p‐mTOR and GAPDH antibodies were purchased from Affinity Biosciences (Cincinnati, USA).

This study was approved by the ethics committee of the General Hospital of Western Theater Command (2022EC2‐ky022). Every effort was made to minimize animal suffering in the study.

### 2.3 Extraction and identification of ucMSC‐Ex

The supernatant was extracted in the third generation cell. UC‐MSCs were centrifuged at 2000 × g and 4°C for 30 min, followed by centrifugation at 10,000 × g and 4°C for 45 min again. The supernatant was collected and centrifuged at 100,000 × g and 4°C for 70 min. Ultracentrifugation was performed at 4°C and 100,000 × g for 70 min. The supernatant was discarded, and the precipitate was resuspended in 200 μL phosphate‐buffered saline (PBS). The isolated partial ucMSC‐Ex were observed using a transmission electron microscope (Hitachi, HT‐7700) and assessed with western blot (WB). Additionally, the size of ucMSC‐Ex was measured using a nanoparticle size analyser.

### 2.4 Experimental grouping and establishment of animal model

Fifty SD rats were randomly divided into four group, namely the control group, TP group, ucMSC‐Ex group (TP+ ucMSC‐Ex group), rapamycin group (TP + ucMSC‐Ex + rapamycin group) and MHY1485 group (TP + ucMSC‐Ex + MHY1485 group). In the control group, rats were fixed after anaesthesia and laparotomy was performed. The pancreas was gently turned over several times with sterile cotton swabs and then closed. Approximately 1 mL normal saline was injected via the tail veins of rats. In the TP group, the modelling approach is presented in a previous study.^4^ In the TP+ ucMSC‐Ex + rapamycin group, a TP model was established for this group. ucMSC‐Ex (10 μg/100 g) were injected via the tail veins of rats. Rapamycin (0.3 mg/mL; 0.2 mg/100 g) was administered by intraperitoneal injection at the end of the procedure described above.

In the TP+ ucMSC‐Ex + MHY1485 group, modelling procedure and the way of exosome injection was as above, and MHY1485 (1.5 mg/mL; 1 mg/100 g) was administered by intraperitoneal injection at the end of the above procedure. All rats were sacrificed after 24 h of modelling, and serum and pancreatic tissue were retained for subsequent experiments. All animal experiments followed the minimize animal suffering. This study was approved by the ethics committee of the General Hospital of Western Theater Command (2022EC2‐ky022).

### 2.5 HE and biochemical detection

Pancreatic tissues from rats in each group were performed to haematoxylin–eosin (HE). The pancreatic oedema, bleeding, cell necrosis and inflammatory cell infiltration were scored by two pathologists blinded to the grouping according to the criteria reported by Schmidt et al.^20^ The average score of 10 high‐power fields was taken as the final score for each slice. Serum amylase, lipase activity and inflammatory factors (interleukin [IL]‐6, IL‐10, tumour necrosis factor‐alpha [TNF‐α] and transforming growth factor [TGF‐β]) were detected using ELISA.

### 2.6 TEM and immunofluorescence

Pancreatic tissue was first fixed and dehydrated, followed by infiltration and embedding. About 60 ~ 90 nm ultrathin sections were prepared using an ultramicrotome and then stained. TEM (JEM‐1400FLASH) was used for image acquisition. Pancreatic tissues were deparaffinized to water by paraffin sections. The sections were immersed in citrate buffer (pH 6.0) and were heated by using microwave oven. Then, goat serum blocking solution was added dropwise, and the primary antibody was incubated. Diamidinophenylindole (DAPI) was added dropwise and incubated for 10 min at room temperature; the sections were blocked using anti‐fluorescence attenuating blocking reagent. The Image‐J analysis system was used to obtain the integrated density and area of all the acquired images, and the mean grey value of each image was calculated, which was recalculated using the mean integrated density of the 2 images to obtain the mean integrated density of each sample.

### 2.7 Real‐time fluorescence quantitative polymerase chain reaction (RT‐qPCR)

Total RNA was isolated and extracted from pancreatic tissue. UV spectrophotometry was used to determine the concentration and purity of the extracted total RNA. RNA was reverse‐transcribed to cDNA. The primer sequences of mTOR, LC3 and p62 were shown in Table 1. The relative mRNA expression levels were calculated by 2^−△△CT^.

**TABLE 1:** RT‐qPCR primer sequences.

Genes	Primer sequences
mTOR	F: gcaacaacctccaggatacactcag
	R: ttccaccagggcttcattgacatc
LC3	F: gagcgagttggtcaagatcatccg
	R: gatgtcagcgatgggtgtggatac
p62	F: ggtgtctgtgagaggacgaggag
	R: tctggtgatggagcctcttactgg
GADPH	F: acagcaacagggtggtggac
	R: tttgagggtgcagcgaactt

### 2.8 Western blot

Protein concentrations were determined with a BCA Protein Quantification Kit. Equal amounts of denatured proteins were separated via sodium dodecyl sulphate–polyacrylamide gel electrophoresis (SDS‐PAGE). The gel face was connected with the negative electrode, and the PVDF membrane was connected with the positive electrode, and the power was switched on for 1–2 h. The PVDF membrane was put into 5% non‐fat milk diluted with TBST buffer into an incubation box and shaken gently for 2 h. The membrane was incubated with the appropriate primary antibody (LC3B 1:2000, mTOR 1:1000, P62 1:10000 p‐mTOR 1:2000, GAPDH 1:50000) at 4°C for 12 h, which followed by incubation with secondary antibodies for 1 h at room temperature. The bands were performed with the GIS Chassis Control Software v2.0, and the results were expressed as relative expression of the protein. (Relative expression of target protein = integrated optical density [IOD]) value of target protein/integrated optical density value of internal reference.

### 2.9 Statistical analyses

The data were performed using SPSS version 25.0 (IBM Corp., Armonk, NY, USA). All of the data were presented as means ± standard deviations (SD) of at least three independent experiments. Univariate comparisons were evaluated by the Student *t*‐test, and multiple comparisons were evaluated by one‐way analysis of variance (ANOVA). Differences were considered significant at *p* < 0.05. Statistical graphs were made by GraphPad Prism (GraphPad Software Corp, San Diego, CA, USA).

## 3 RESULTS

### 3.1 High‐throughput sequencing results and analysis

**FIGURE 1:**
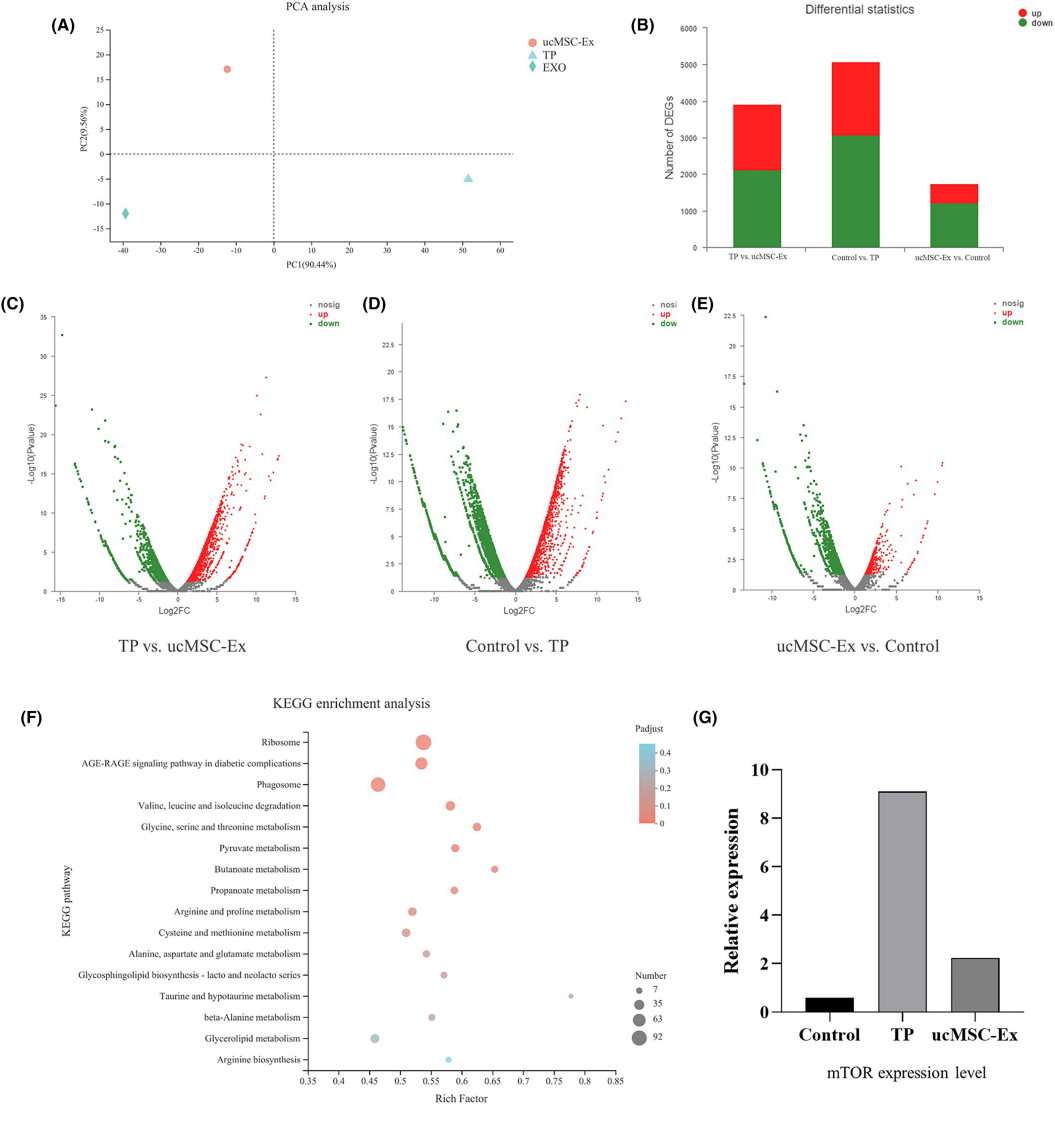
Whole genome high‐throughput sequencing of pancreatic tissue. (A) PCA of pancreatic tissues from the three groups. (B) Quantitative analysis of expression of differential genes. (C) Differential gene expression volcano plot of TP and ucMSC‐Ex groups. (D) Differential gene expression volcano plot of TP and control groups. (E) Differential gene expression volcano plot of control and ucMSC‐Ex groups. (F) KEGG enrichment analysis: Bubble chart. (G) Expression of mTOR.

We have uploaded the sequencing data to the Gene Expression Omnibus (GEO) database, numbered GSE214370. The results of principal component analysis (PCA) showed high heterogeneity among samples, which suggested successful modelling and reasonable experimental design (Figure 1A). We analysed the differences of transcripts between groups. The results showed that 3911 differential genes were screened in TP and ucMSC‐Ex groups, including 1785 up‐regulated genes and 2126 down regulated genes (Figure 1C). Similarly, 5083 differential genes were screened in the control and TP groups, including 2004 up‐regulated genes and 3079 down regulated genes (Figure 1D). A total of 1739 differential genes were screened in the control and ucMSC‐Ex groups, including 516 up‐regulated genes and 1223 down regulated genes (Figure 1E). The statistical analysis of differential gene expression is shown in Figure 1B.

Kyoto Encyclopedia of Genes and Genomes (KEGG) enrichment analysis showed that 63 autophagy‐related genes had significant differences, accounting for 45% of the total genes. According to the adjusted P value, the significance of autophagy is in the third place (Figure 1F). Among them, the gene expression of mTOR increased in TP group and decreased in ucMSC‐Ex group (Figure 1G).

### 3.2 Extraction and identification of exosomes

**FIGURE 2:**
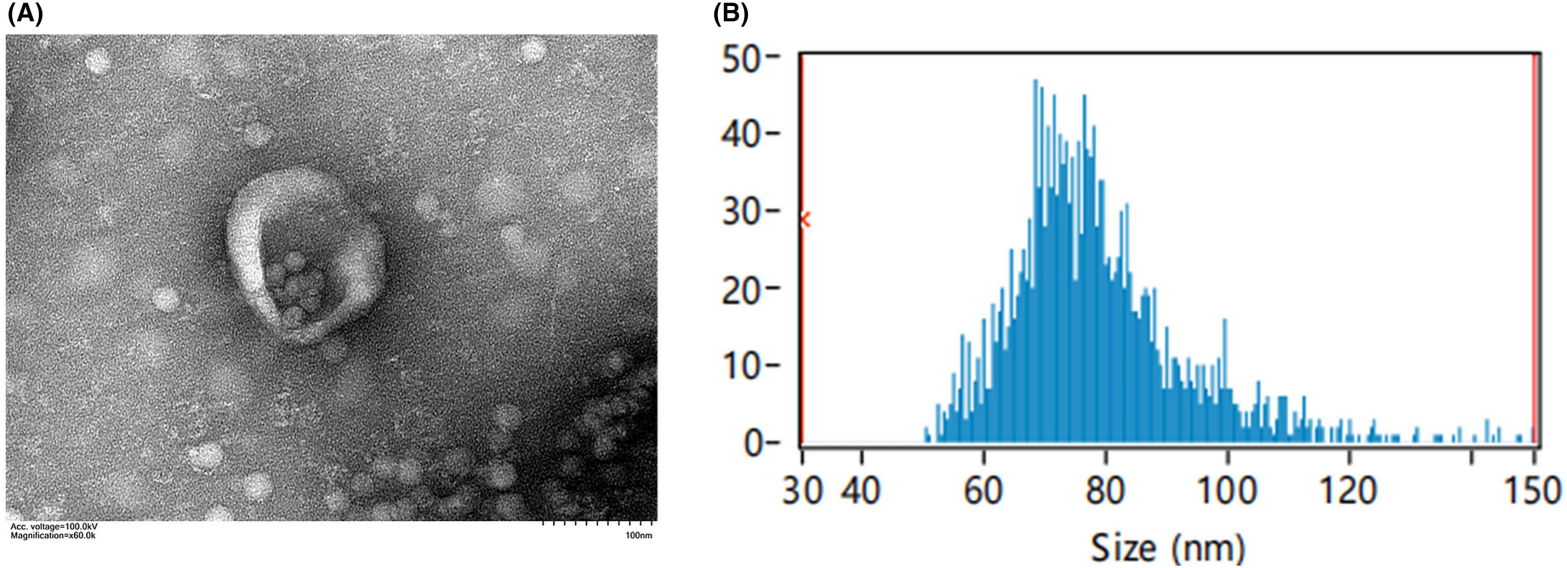
Extraction and identification of ucMSC‐Ex. (A) TEM of ucMSC‐Ex. (B) Nanoparticle tracking analysis of ucMSC‐Ex.

Transmission electron microscope (TEM) was used to identify the shape of the exocrine body as a vesicular structure (Figure 2A). The average particle size of exosomes is 78.51 nm, and the concentration is 2.89*10^9^ Particles/mL. It indicates that the exosomes were successfully extracted and identified (Figure 2B).

### 3.3 Analysis of pathological injury of pancreatic tissue and biochemical detection

**FIGURE 3:**
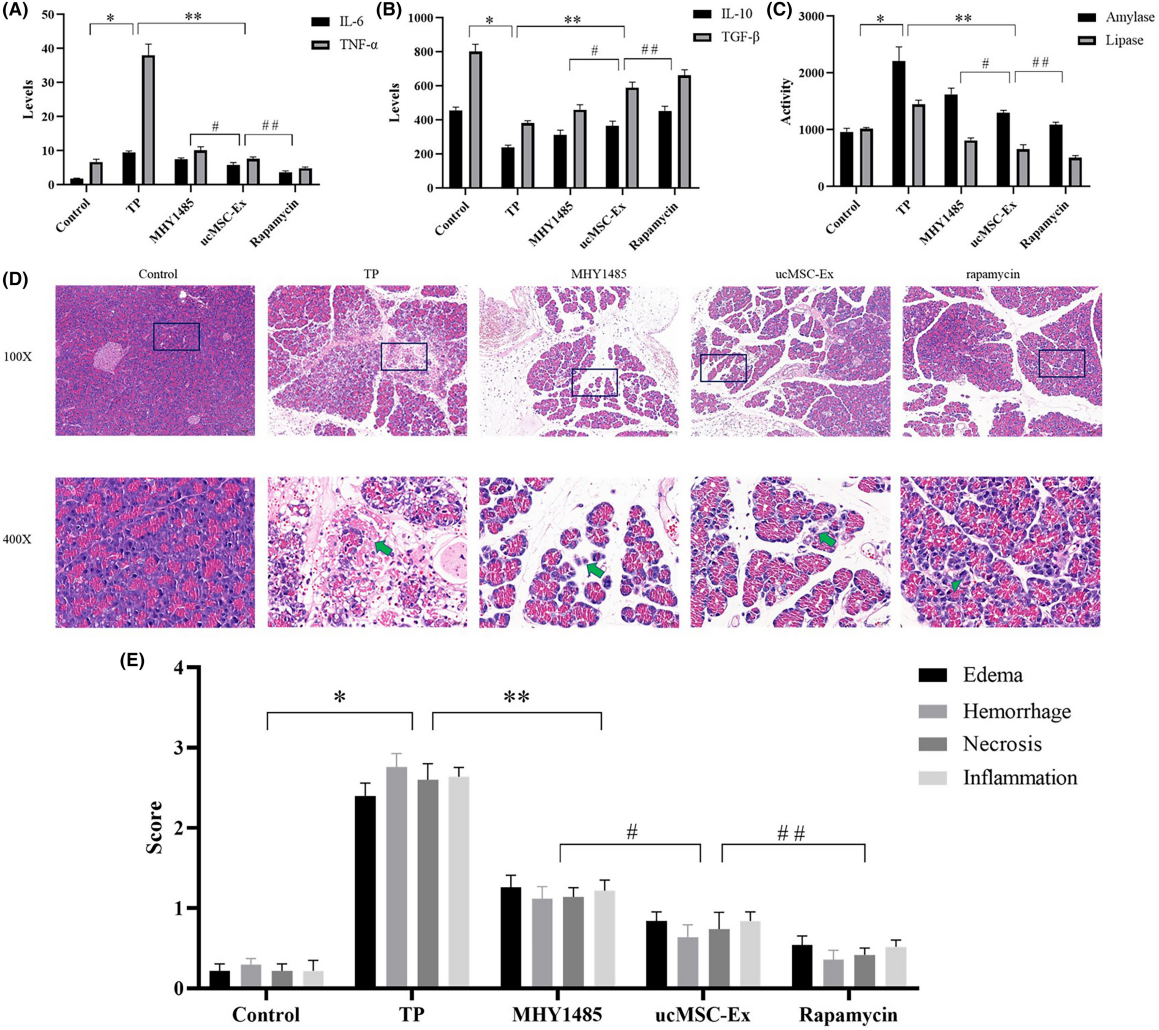
ELISA detection of serum markers. (A) Pro‐inflammatory factor (IL‐6, TNF‐α). (B) Anti‐inflammatory factor (IL‐10, TGF‐β). (C) Serum enzymology (amylase, lipase). (D) Pathological injury of pancreatic tissue: HE stain. (E) The pancreatic oedema, bleeding, cell necrosis and inflammatory cell infiltration scores. Green arrow indicates necrosis. *Control vs. TP, *p* < 0.05; **TP vs. ucMSC‐Ex, *p* < 0.05; ^#^ucMSC‐Ex vs. MHY1485, *p* < 0.05; ^##^ucMSC‐Ex vs. Rapamycin, *p* < 0.05.

In the TP group, pro‐inflammatory factors (IL‐6 and TNF‐α) were significantly increased and anti‐inflammatory (IL‐10 and TGF‐β) factors significantly decreased. In the MHY1485, ucMSC‐Ex and Rapamycin groups, the pro‐inflammatory factors increased in turn, while the anti‐inflammatory factors decreased in turn. The expression of lipase and amylase exhibited a trend consistent with pro‐inflammatory factors. The above differences were statistically significant (Figure 3A–C).

In the control group, there was no obvious necrosis, bleeding and other pathological damage. In other groups, there were different degrees of damage. In the TP group, the pancreatic tissue structure was confused, the boundary was unclear, and obvious haemorrhage and inflammatory cell infiltration could be seen. In MHY1485 group, the pancreatic structure was clear, a few acinar cells were necrotic and degenerated, and no obvious haemorrhage was found. In the ucMSC‐Ex group, the gap between acinar cells was slightly widened, and the stroma was slightly oedematous with inflammatory cell infiltration. In Rapamycin group, interstitial haemorrhage was slight, and some acinar cells were necrotic and degenerative (Figure 3D). The pathological scores of TP group were significantly different from those of control group (Figure 3E). The pathological scores of MHY1485 group, ucMSC‐Ex and Rapamycin groups decreased in turn, with statistical difference. In addition, the degree of pathological damage in the three above groups was significantly improved compared with that in the TP group.

### 3.4 Analysis of autophagy in pancreatic tissue

**FIGURE 4:**
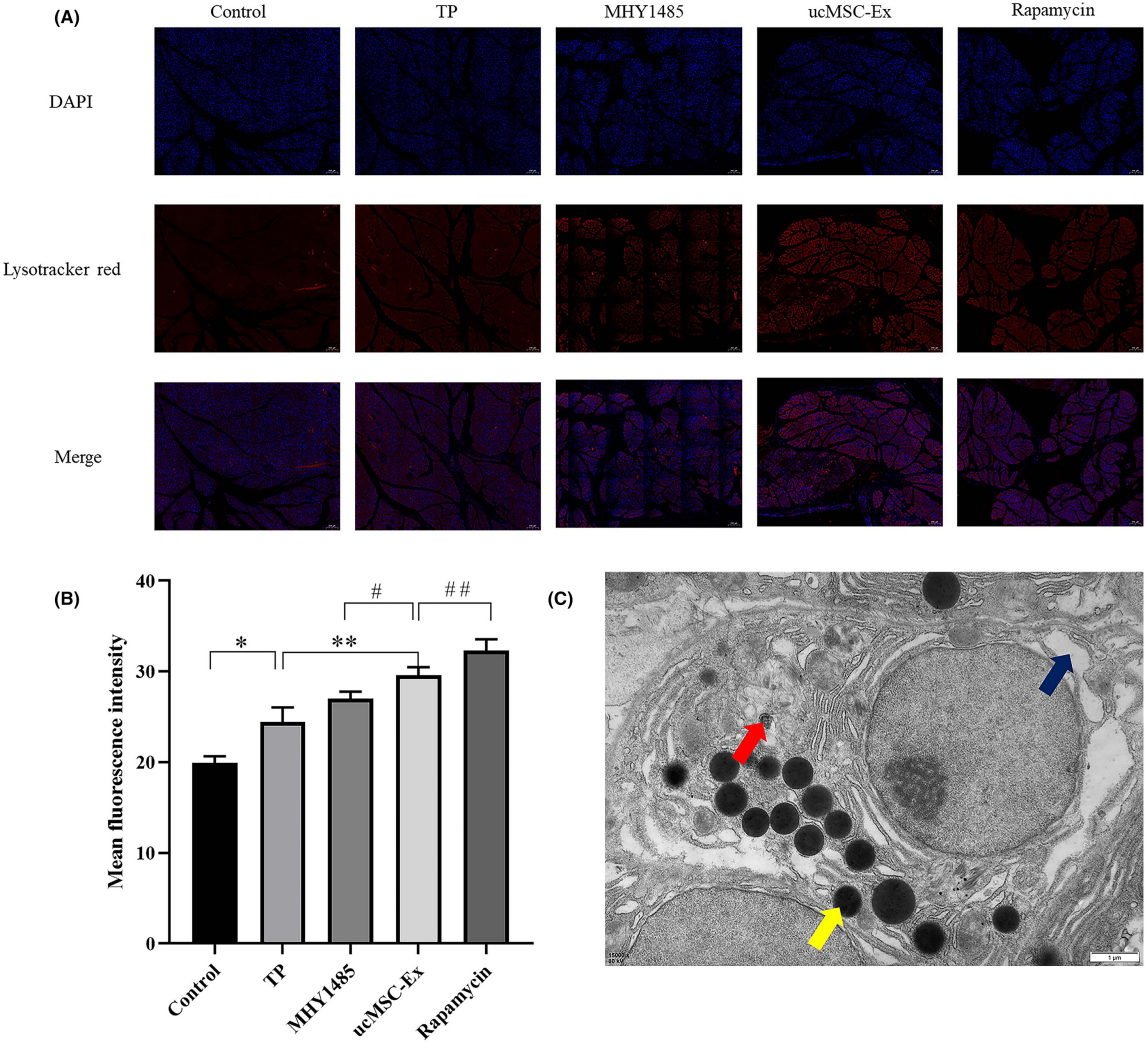
Qualitative and quantitative detection of autophagy in pancreatic tissue. (A) Immunofluorescence imaging. (B) Quantitative analysis of fluorescence intensity. (C) TEM of autophagosome. The red arrow indicates autophagosome; the blue arrow indicates swollen mitochondria; and the yellow arrow indicates zymogen particles. *Control vs. TP, *p* < 0.05; **TP vs. ucMSC‐Ex, *p* < 0.05; ^#^ucMSC‐Ex vs. MHY1485, *p* < 0.05; ^##^ucMSC‐Ex vs. Rapamycin, *p* < 0.05.

In the control group, no obvious effect of autophagy was observed. Different degrees of autophagy effect were observed in the treatment groups. Compared to the TP group, the intensity of the autophagic effect was significantly higher in the ucMSC‐Ex group. Using MHY1485, the fluorescence intensity decreased significantly. Whereas the fluorescence intensity significantly increased after being treated with paromomycin. Differences were considered statistically significant (Figure 4A,B). TEM revealed that pancreatic acinar cells had abundant rough endoplasmic reticulum and zymogen granules, and autophagosomes were observed. It suggested that autophagy occurred in acinar cells (Figure 4C).

### 3.5 Analysis of autophagy‐related genes and protein expression profiles

**FIGURE 5:**
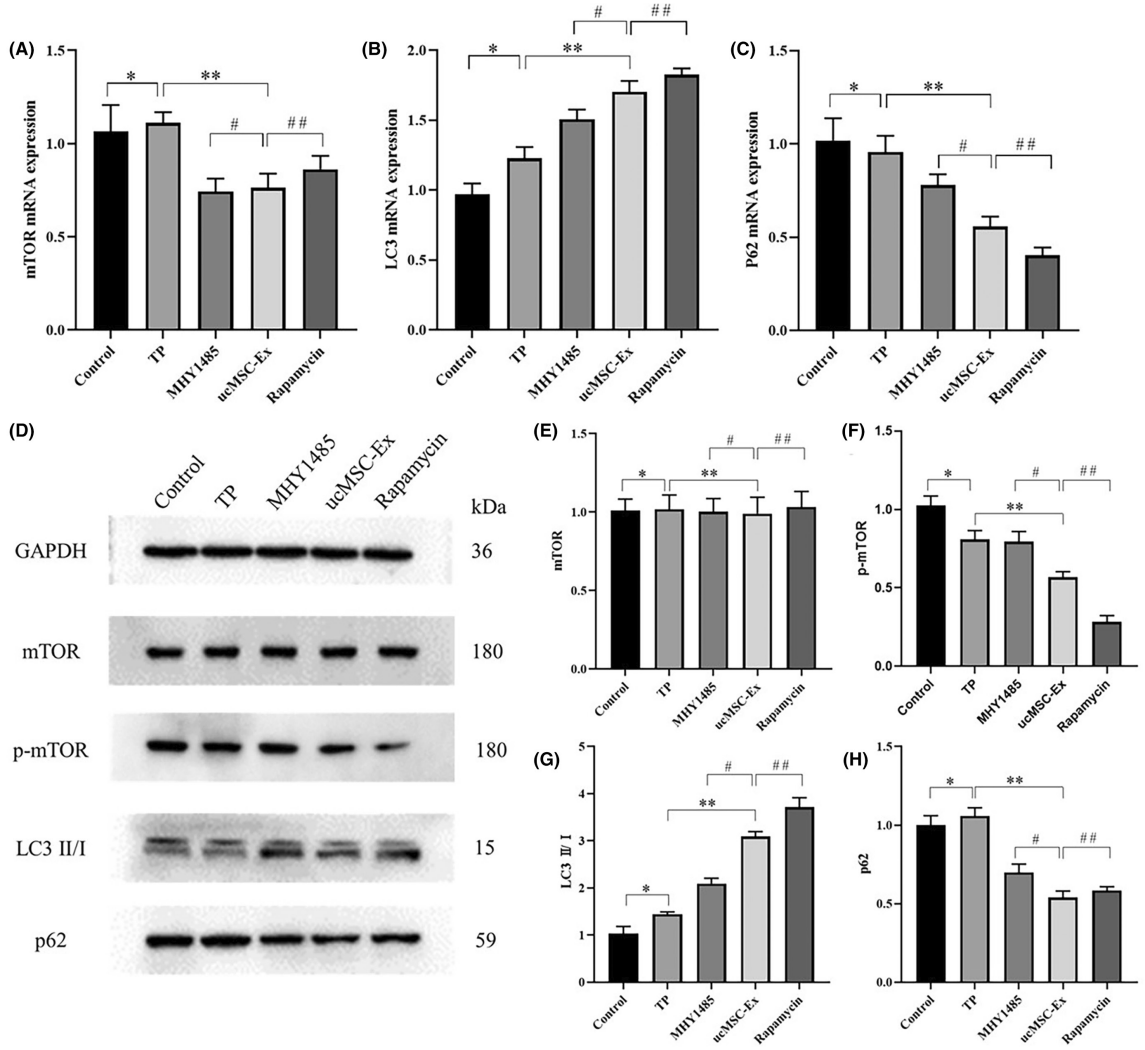
(A) RT‐qPCR: The expression of mTOR mRNA. (B) The expression of LC3 mRNA. (C) The expression of P62 mRNA. (D) Western blot: Protein band. (E) The expression of mTOR. (F) The expression of p‐mTOR. (G) The expression of LC3 II/I. (H) The expression of P62. *Control vs. TP, *p* < 0.05; **TP vs. ucMSC‐Ex, *p* < 0.05; ^#^ucMSC‐Ex vs. MHY1485, *p* < 0.05; ^##^ucMSC‐Ex vs. Rapamycin, *P* < 0.05.

In the MHY1485, ucMSC‐Ex and Rapamycin groups, mTOR expression levels were significantly decreased. Autophagy‐related LC3 showed an upward trend. On the contrary, P62 showed a decreasing trend. The above differences were statistically significant (Figure 5A–C).

Western blot showed that p‐mTOR expression levels were significantly decreased in MHY, ucMSC‐Ex and Rapamycin groups. The ratio of LC3 II/I showed an increasing trend. The expression levels of P62 showed an opposite trend. The above differences were statistically significant (Figure 5D–H).

## 4 DISCUSSION

**FIGURE 6:**
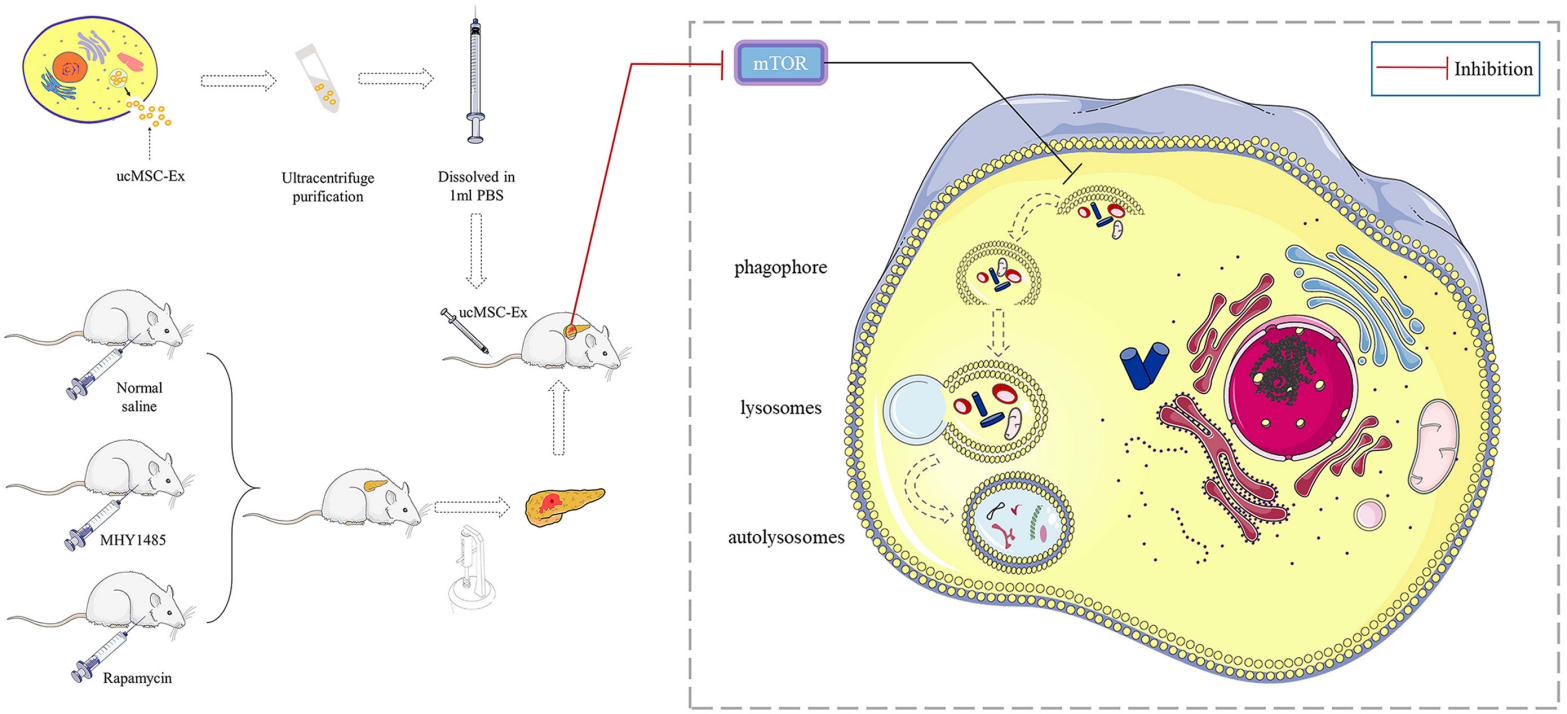
Article structure schematic.

Traumatic pancreatitis is a class of inflammatory diseases of the pancreas, which is highly susceptible to patient death and the lack of effective therapeutic choices. It is necessary to increase researches for treatment of TP.^21^ However, the ability of examine functional biological questions in TP has been hampered owing to the lack of suitable animal models and stable trauma control devices. In addition, the unclear expression level of genes related to post‐traumatic stress and repair presents are additional challenges in understanding the development of this disease. Our previous study established a stable TP model and confirmed ucMSC‐Ex exerts reparative effects on TP.^4^, ^5^, ^19^ In this study, we further dissected the transcriptomics after pancreatic trauma and ucMSC‐Ex therapy by high‐throughput sequencing. KEGG enrichment analysis and differential analysis identified that autophagy played a crucial role in the pathophysiological process of TP and ucMSC‐Ex therapy. The wide variation in the amount of mTOR expressed as the core of autophagy regulation suggested that ucMSC‐Ex may exert therapeutic effects by influencing their function. Our experiment supported this view by activating and inhibiting mTOR to influence the therapeutic efficacy of ucMSC‐Ex on TP (Figure 6).

Autophagy is an adaptive response activated under stress to maintain cellular energy homeostasis through the autolysosomal degradation pathway and clear damaged organelles.^22^, ^23^ In this process, intracellular components are targeted for elimination by lysosome dependent recycling mechanism.^24^, ^25^ Recent researches have emphasized the importance of autophagy in the occurrence and severity of acute pancreatitis.^15^, ^26^, ^27^ The key event in the development of acute pancreatitis is the overactivation of trypsinogen within the pancreas, whereas activated autophagy will clear activated trypsinogen and damaged organelles with high efficiency.^28^ Further, much attention has been paid to the regulation of autophagy during the development of pancreatitis.^29^ In the present study, a rat model of traumatic pancreatitis with a fixed range and extent of injury was used to explore the specific mechanisms by which ucMSC‐Ex exert therapeutic effects through modulation of autophagy. Firstly, we first performed whole transcriptome sequencing and found that the expression of autophagy‐related genes was significantly different by KEGG enrichment analysis.

Autophagy is regulated by several pathways, such as depletion of TX‐2 increases Atg16L1 binding to inclusion proteins, which leads to impaired autophagic flux and increased severity of AP.^30^ An experiment in a pancreatitis model which was constructed in ATG5 knockout mice reported that autophagy was activated in these animals resulting in a reduction in the severity of pancreatitis.^31^ Further quantitative analysis on the expression level of autophagy‐related genes enriched by KEGG showed that the expression level of mTOR was greatly increased after trauma, and recovered after ucMSC‐Ex treatment. mTOR is a serine / threonine protein kinase, and activation of the mTOR pathway is involved in many aspects of molecular and cellular biology. For autophagy effects, the initiating factor lies in the activation of ULK1 and AMBRA1 complexes following AMPK activation and mTOR inhibition.^32^ The regulation of this progress to affect autophagy has been well‐established applied to in vivo and *vitro* experiments. As a classical upstream regulator of autophagy, inhibition of mTOR has been shown to exert protective effects in chronic pancreatitis (CP).^33^

However, the effect in traumatic pancreatitis has not been fully studied. In order to analyse the influence of ucMSC‐Ex on the expression of mTOR on pancreatitis, we used rapamycin and MHY1485 to regulate mTOR. Subsequent histological examination (HE) detection of pancreatic tissue and quantitative detection of serum inflammatory factors found that traumatic pancreatitis in rats after ucMSC‐Ex treatment had been significantly alleviated. On this basis, inhibition of mTOR could play a more significant therapeutic effect. Especially, serum levels of amylase and lipase were greatly reduced, suggesting that mTOR inhibition confers significant suppression of the ‘waterfall effect’ in pancreatitis. Further immunofluorescence staining revealed that the amount of red fluorescence expression in pancreatic tissue was increased after exosome treatment, which suggested autophagy was significantly enhanced and the autophagosomes were observed by electron microscopy. These confirmed the above trends.

The mTOR pathway is closely related to the proliferation, differentiation, apoptosis, motility, metabolism and autophagy of cells, and the regulation of this pathway contributes to the precision treatment of pancreatic diseases.^34^ To determine the gene expression levels and protein expression amounts of mTOR and autophagy, we performed RT‐qPCR and WB assays, and found that the inhibition of mTOR directly led to the enhancement of autophagic flux. More specifically, significant decrease in phosphorylated mTOR protein. As a result, the ULK1‐mediated catabolic programme was activated, leading directly to the enhancement of autophagy. An experimental finding consisting of three different kinds models of AP with increased serum AMY and PNLIP revealed a link between impaired autophagy and the release of digestive enzymes into the circulation.^12^ Another study in this area similarly found that perturbing the basal pancreatic acinar cell autophagy effect would directly exacerbate inflammatory damage and produce a higher likelihood of severity.^35^ Our study supported this widely observed phenomenon.

As discussed, the prognosis of pancreatitis is closely related to the effect of autophagy. On the basis of our demonstration that ucMSC‐Ex exerts therapeutic TP by enhancing autophagy via inhibiting mTOR, the mechanism by which ucMSC‐Ex achieve regulatory effects remain unclear. The effects of non‐coding RNA enriched in ucMSC‐Ex on signalling processes have been observed, but the specific mechanisms still need further verification. Future studies will build on the comprehensive sequencing of RNA carried by exosomes to predict and experimentally validate specific non‐coding RNA playing such a role.

## 5 CONCLUSION

We revealed an important role of mTOR, which may exert therapeutic effects by regulating acinar cell autophagy after TP. Therefore, ucMSC‐Ex may repress the expression of mTOR enhancing autophagy as an effective therapy for TP, which may be a potential drug development direction for TP patients.

## AUTHOR CONTRIBUTIONS

**Zhirong Zhao:** Data curation (lead); methodology (lead); writing – original draft (lead). **Li Han:** Formal analysis (lead); investigation (supporting); methodology (supporting). **Mei Xin:** Investigation (supporting); methodology (supporting). **Lichen Zhou:** Investigation (supporting); methodology (supporting). **Kexin Jiang:** Investigation (supporting); methodology (supporting). **Qian Huang:** Investigation (supporting); methodology (supporting). **Ruiwu Dai:** Conceptualization (lead); funding acquisition (lead); supervision (lead); writing – review and editing (lead).

## FUNDING INFORMATION

This work was supported by the Hospital Management of the General Hospital of Western Theater Command [grant numbers 2021‐XZYG‐B16]; the key research and development programme of Sichuan Provincial Science and Technology Department [grant number 2022YFS0195]; the Pancreatic injury and repair Key laboratory of Sichuan Province [grant number 41732152]; and The National Natural Science Foundation of China [grant numbers 82070579].

## CONFLICT OF INTEREST STATEMENT

The authors declare that they have no competing interests.

## Data Availability

Data availability original reagents are available upon request. The datasets generated and/or analysed during the current study are available in the (GEO) repository, (https://www.ncbi.nlm.nih.gov/geo/query/acc.cgi?acc=GSE214370).

## References

[1] Sharbidre KG , Galgano SJ , Morgan DE . Traumatic pancreatitis. Abdom Radiol (NY). 2020;45:1265‐1276. doi:10.1007/s00261-019-02241-7 31576413

[2] Dai R , Chen G , Huang Z , et al. Establishment and characteristics of an animal model for isolated pancreatic trauma. J Trauma Acute Care Surg. 2012;73:648‐653. doi:10.1097/TA.0b013e318250ad07 23007015

[3] Zhu Q , Lin X , Liu X , et al. Dynamic changes of proteasome and protective effect of bortezomib, a proteasome inhibitor, in mice with acute pancreatitis. Biochem Biophys Res Commun. 2018;505:126‐133. doi:10.1016/j.bbrc.2018.09.066 30236985

[4] Han L , Zhao Z , Chen X , et al. Human umbilical cord mesenchymal stem cells‐derived exosomes for treating traumatic pancreatitis in rats. Stem Cell Res Ther. 2022;13:221. doi:10.1186/s13287-022-02893-1 35619158 PMC9137180

[5] Zhirong Z , Li H , Yiqun H , et al. Enhancing or inhibiting apoptosis? The effects of ucMSC‐ex in the treatment of different degrees of traumatic pancreatitis. Apoptosis. 2022;27:521‐530. doi:10.1007/s10495-022-01732-1 35612769

[6] Han L , Zhao Z , Yang K , et al. Application of exosomes in the diagnosis and treatment of pancreatic diseases. Stem Cell Res Ther. 2022;13:153. doi:10.1186/s13287-022-02826-y 35395948 PMC8994331

[7] Kapuy O , Vinod PK , Bánhegyi G . mTOR inhibition increases cell viability via autophagy induction during endoplasmic reticulum stress ‐ An experimental and modeling study. FEBS Open Bio. 2014;4:704‐713. doi:10.1016/j.fob.2014.07.006 PMC4141208 25161878

[8] Adler G , Rohr G , Kern HF . Alteration of membrane fusion as a cause of acute pancreatitis in the rat. Dig Dis Sci. 1982;27:993‐1002. doi:10.1007/bf01391745 7140496

[9] Aho HJ , Nevalainen TJ , Havia VT , Heinonen RJ , Aho AJ . Human acute pancreatitis: a light and electron microscopic study. Acta pathologica, microbiologica, et immunologica Scandinavica. Section A, Pathology. 1982;90:367‐373. 7148455

[10] Gukovskaya AS , Gukovsky I . Autophagy and pancreatitis. Am J Physiol Gastrointest Liver Physiol. 2012;303:G993‐G1003. doi:10.1152/ajpgi.00122.2012 22961802 PMC3517664

[11] Gukovskaya AS , Gukovsky I , Algül H , Habtezion A . Autophagy, inflammation, and immune dysfunction in the pathogenesis of pancreatitis. Gastroenterology. 2017;153:1212‐1226. doi:10.1053/j.gastro.2017.08.071 28918190 PMC6338477

[12] Mareninova OA , Jia W , Gretler SR , et al. Transgenic expression of GFP‐LC3 perturbs autophagy in exocrine pancreas and acute pancreatitis responses in mice. Autophagy. 2020;16:2084‐2097. doi:10.1080/15548627.2020.1715047 31942816 PMC7595606

[13] Zhao SP , Yu C , Xiang KM , Yang MS , Liu ZL , Yang BC . miR‐375 inhibits autophagy and further promotes inflammation and apoptosis of acinar cells by targeting ATG7. Pancreas. 2020;49:543‐551. doi:10.1097/mpa.0000000000001536 32282768

[14] Jiang X , Zheng YW , Bao S , et al. Drug discovery and formulation development for acute pancreatitis. Drug Deliv. 2020;27:1562‐1580. doi:10.1080/10717544.2020.1840665 33118404 PMC7598990

[15] Mei Q , Zeng Y , Huang C , et al. Rapamycin alleviates hypertriglyceridemia‐related acute pancreatitis via restoring autophagy flux and inhibiting endoplasmic reticulum stress. Inflammation. 2020;43:1510‐1523. doi:10.1007/s10753-020-01228-7 32642911

[16] Wang N , Zhang F , Yang L , et al. Resveratrol protects against L‐arginine‐induced acute necrotizing pancreatitis in mice by enhancing SIRT1‐mediated deacetylation of p53 and heat shock factor 1. Int J Mol Med. 2017;40:427‐437. doi:10.3892/ijmm.2017.3012 28586010 PMC5504992

[17] Li D , Li Y , Li M , et al. Population genomics identifies patterns of genetic diversity and selection in chicken. BMC Genomics. 2019;20:263. doi:10.1186/s12864-019-5622-4 30940068 PMC6446315

[18] Song JJ , Go YY , Lee JK , et al. Transcriptomic analysis of tobacco‐flavored E‐cigarette and menthol‐flavored E‐cigarette exposure in the human middle ear. Sci Rep. 2020;10:20799. doi:10.1038/s41598-020-77816-2 33247188 PMC7699635

[19] Li H , Zhirong Z , Shibo Z , et al. The effects of umbilical cord mesenchymal stem cells on traumatic pancreatitis in rats. Dig Dis Sci. 2022;68:147‐154. doi:10.1007/s10620-022-07493-w 35430701

[20] Schmidt J , Rattner DW , Lewandrowski K , et al. A better model of acute pancreatitis for evaluating therapy. Ann Surg. 1992;215:44‐56. doi:10.1097/00000658-199201000-00007 1731649 PMC1242369

[21] Girard E , Abba J , Cristiano N , et al. Management of splenic and pancreatic trauma. J Visc Surg. 2016;153:45‐60. doi:10.1016/j.jviscsurg.2016.04.005 27402320

[22] Levine B , Kroemer G . Autophagy in the pathogenesis of disease. Cell. 2008;132:27‐42. doi:10.1016/j.cell.2007.12.018 18191218 PMC2696814

[23] Morishita H , Mizushima N . Diverse cellular roles of autophagy. Annu Rev Cell Dev Biol. 2019;35:453‐475. doi:10.1146/annurev-cellbio-100818-125300 31283377

[24] Dewi FRP , Jiapaer S , Kobayashi A , et al. Nucleoporin TPR (translocated promoter region, nuclear basket protein) upregulation alters MTOR‐HSF1 trails and suppresses autophagy induction in ependymoma. Autophagy. 2021;17:1001‐1012. doi:10.1080/15548627.2020.1741318 32207633 PMC8078762

[25] Klionsky DJ , Abdel‐Aziz AK , Abdelfatah S , et al. Guidelines for the use and interpretation of assays for monitoring autophagy (4th edition)^1^ . Autophagy. 2021;17:1‐382. doi:10.1080/15548627.2020.1797280 33634751 PMC7996087

[26] Gukovsky I , Li N , Todoric J , Gukovskaya A , Karin M . Inflammation, autophagy, and obesity: common features in the pathogenesis of pancreatitis and pancreatic cancer. Gastroenterology. 2013;144:1199‐1209.e1194. doi:10.1053/j.gastro.2013.02.007 23622129 PMC3786712

[27] Wang XD , Yu WL , Sun Y . Activation of AMPK restored impaired autophagy and inhibited inflammation reaction by up‐regulating SIRT1 in acute pancreatitis. Life Sci. 2021;277:119435. doi:10.1016/j.lfs.2021.119435 33781829

[28] Mareninova OA , Hermann K , French SW , et al. Impaired autophagic flux mediates acinar cell vacuole formation and trypsinogen activation in rodent models of acute pancreatitis. J Clin Invest. 2009;119:3340‐3355. doi:10.1172/jci38674 19805911 PMC2769194

[29] Voronina S , Chvanov M , de Faveri F , et al. Autophagy, acute pancreatitis and the metamorphoses of a trypsinogen‐activating organelle. Cell. 2022;11:2514. doi:10.3390/cells11162514 PMC9406838 36010591

[30] Dolai S , Liang T , Orabi AI , et al. Pancreatitis‐induced depletion of syntaxin 2 promotes autophagy and increases basolateral exocytosis. Gastroenterology. 2018;154:1805‐1821.e1805. doi:10.1053/j.gastro.2018.01.025 29360461 PMC6461447

[31] Hashimoto D , Ohmuraya M , Hirota M , et al. Involvement of autophagy in trypsinogen activation within the pancreatic acinar cells. J Cell Biol. 2008;181:1065‐1072. doi:10.1083/jcb.200712156 18591426 PMC2442206

[32] Nazio F , Strappazzon F , Antonioli M , et al. mTOR inhibits autophagy by controlling ULK1 ubiquitylation, self‐association and function through AMBRA1 and TRAF6. Nat Cell Biol. 2013;15:406‐416. doi:10.1038/ncb2708 23524951

[33] Kong L , Xu X , Zhang H , et al. Human umbilical cord‐derived mesenchymal stem cells improve chronic pancreatitis in rats via the AKT‐mTOR‐S6K1 signaling pathway. Bioengineered. 2021;12:1986‐1996. doi:10.1080/21655979.2021.1928441 34047671 PMC8806739

[34] LoRusso PM . Inhibition of the PI3K/AKT/mTOR pathway in solid tumors. J Clin Oncol. 2016;34:3803‐3815. doi:10.1200/jco.2014.59.0018 27621407 PMC6366304

[35] Halangk W , Lerch MM , Brandt‐Nedelev B , et al. Role of cathepsin B in intracellular trypsinogen activation and the onset of acute pancreatitis. J Clin Invest. 2000;106:773‐781. doi:10.1172/jci9411 10995788 PMC381392

